# Supplementary material for: Iron Elution from Iron and Steel Slag Using Bacterial Complex Identified from the Seawater
Source: Materials (Basel). 2021 Mar 17;14(6):1477. doi: 10.3390/ma14061477 (PMC8002712; doi:10.3390/ma14061477)
Supplement: Supplementary file 1 [file materials-14-01477-s001.pdf]

Supporting information

Iron Elution from Iron and Steel Slag Using Bacterial Complex Identified from the Seawater

Hidenori Tsukidate [1,†], Seika Otake [1,†], Yugo Kato [1], Ko Yoshimura [2], Masafumi Kitatsuji [3], Etsuro Yoshimura [1,4] and Michio Suzuki [1,*]

1 Department of Applied Biological Chemistry, Graduate School of Agriculture, The University of Tokyo, 1-1-1 Yayoi, Bunkyo, Tokyo 113-8657, Japan; honhanhenhin345@gmail.com (H.T.); seikaotake123@gmail.com (S.O.); katoh.yugo@gmail.com (Y.K.) ; yoshimura.sb9.koh@jp.nipponsteel.com (E.Y.)
2 Advanced Technology Research Laboratories, Nippon Steel, 20-1 Shintomi, Futtsu, Chiba 2930011, Japan; etsuroyoshimura@g.ecc.u-tokyo.ac.jp
3 School of Food Industrial Sciences, Miyagi University, 2-2-1, Hatatate, Tauhaku, Sendai, Miyagi 982-0215, Japan; kitatsuj@myu.ac.jp
4 Department of Liberal Arts, The Open University of Japan, 2-11 Wakaba, Mishima-ku, Chiba 261-8586, Japan
* Correspondence: amichio@g.ecc.u-tokyo.ac.jp
† These authors contributed equally to this work.

Table S1. Components of modified Marine Broth 2216.

Modified Marine Broth 2216	
**NaCl**	19.45 g
$MgCl_2 \cdot 6H_2O$	18.8 g
Na_2SO_4	3.24 g
$CaCl_2 \cdot 2H_2O$	2.4 g
KCl	0.55 g
$NaHCO_3$	0.16 g
KBr	0.08 g
$SrCl_2 \cdot 6H_2O$	57.1 mg
H_3BO_3	22.0 mg
$Na_2SiO_3 \cdot 9H_2O$	9.3 mg
NaF	2.4 mg
NH_4NO_3	1.6 mg
$Na_2HPO_4 \cdot 12H_2O$	20.2 mg
sodium citrate	0.12 g
$FeCl_3 \cdot 6H_2O$	0.11 g
Bacto peptone	5.0 g
Bacto yeast extract	1.0 g
**Agar**	15 g

Table S2. Components of Non-Iron modified Marine Broth 2216.

Non-Iron Modified Marine Broth 2216	
**NaCl**	19.45 g
$MgCl_2 \cdot 6H_2O$	18.8 g
Na_2SO_4	3.24 g
$CaCl_2 \cdot 2H_2O$	2.4 g
KCl	0.55 g
$NaHCO_3$	0.16 g
KBr	0.08 g
$SrCl_2 \cdot 6H_2O$	57.1 mg
H_3BO_3	22.0 mg
$Na_2SiO_3 \cdot 9H_2O$	9.3 mg
NaF	2.4 mg
NH_4NO_3	1.6 mg
$Na_2HPO_4 \cdot 12H_2O$	20.2 mg
Bacto peptone	5.0 g
Bacto yeast extraxt	1.0 g
HEPES	2.38 g

Table S3. The relative iron elution and biofilm synthesis of each strain. ○: large amount of biofilm, △: middle amount of biofilm, ×: small amount of biofilm.

Strain	Relative Iron Elution	Biofilm	Strain	Relative Iron Elution	Biofilm
TO1	6.58	×	TO20	2.07	×
TO2	5.08	×	TO21	2.05	×
TO3	4.86	×	TO22	1.96	×
TO4	4.35	×	TO23	1.77	△
TO5	3.98	×	TO24	1.72	×
TO6	3.95	△	TO25	1.69	×
TO7	3.79	○	TO26	1.63	×
TO8	3.66	△	TO27	1.53	×
TO9	3.52	×	TO28	1.46	×
TO10	3.50	×	TO29	1.43	△
TO11	3.09	×	TO30	1.43	×
TO12	2.83	×	TO31	1.41	×
TO13	2.81	×	TO32	1.41	×
TO14	2.73	×	TO33	1.38	×
TO15	2.71	×	TO34	1.30	△
TO16	2.56	×	TO35	1.28	△
TO17	2.37	×	TO36	1.26	△
TO18	2.20	△	TO37	1.14	×
TO19	2.16	×	TO38	1.11	×
			negative control	1.00	×

Table S4. Accession numbers of 16S rRNA for phylogenetic tree of *Sulfitobacter* genus in Fig. 3A.

Type Strain	Accession No.
Sulfitobacter pontiacus	Y13155
Sulfitobacter litoralis	DQ097527
Sulfitobacter brevis	DQ975633
Sulfitobacter marinus	DQ683726
Sulfitobacter mediterraneus	Y17387
Sulfitobacter porphyrae	AB758574
Sulfitobacter donghicola	EF202614
Sulfitobacter dubius	AY180102
Oceanibulbus indolifex	DQ915614
Sulfitobacter delicatus	AY180103

Table S5. Accession numbers of 16S rRNA for phylogenetic tree of *Pseudomonas* genus in Fig. 3B.

Type Strain	Accession No.
Pseudomonas marincola	AB301071
Pseudomonas segetis	AY770691
Pseudomonas borbori	AM114527
Pseudomonas cuatrocienegasensis	EU791281
Pseudomonas prosekii	JN814372
Pseudomonas taeanensis	FJ424813
Pseudomonas composti	FN429930
Pseudomonas anguilliseptica	X99540
Pseudomonas guineae	AM491810
Pseudomonas peli	AM114534

Table S6. Accession numbers of 16S rRNA for phylogenetic tree of *Pseudoalteromonas* genus in Fig. 8.

Type Strain	Accession No.
Pseudoalteromonas piscicida	AB090232
Pseudoalteromonas espejiana	X87143
Pseudoalteromonas marina	AY563031
Pseudoalteromonas aurantia	X82135
Pseudoalteromonas byunsanensis	DQ011289
Pseudoalteromonas xiamenensis	JN188399
Pseudoalteromonas maricaloris	AF144036
Pseudoalteromonas flavipulchra	AF297958
Pseudoalteromonas ulvae	AF172987
Pseudoalteromonas tunicata	Z25522

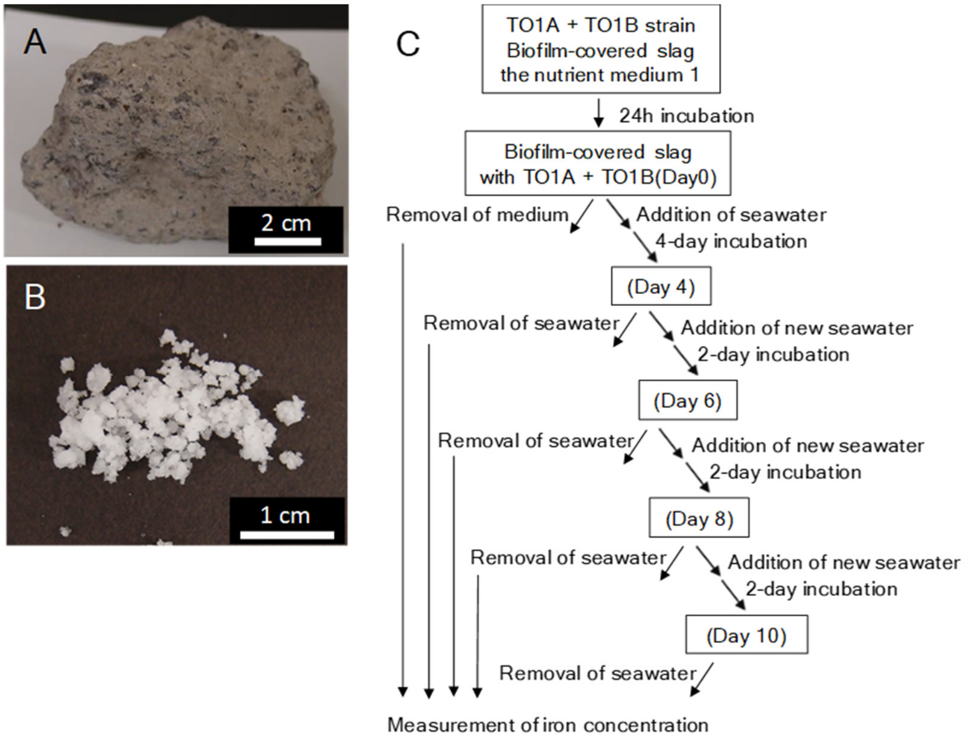

Figure S1. Biofilm and iron elution of CSMS in seawater. (A) Steel slag hydrated matrix (B) Artificial biofilm formed by agarose. (C) The procedure of the experiment for the iron elution capacity of TO1 in seawater. The iron concentration was measured 5 times.

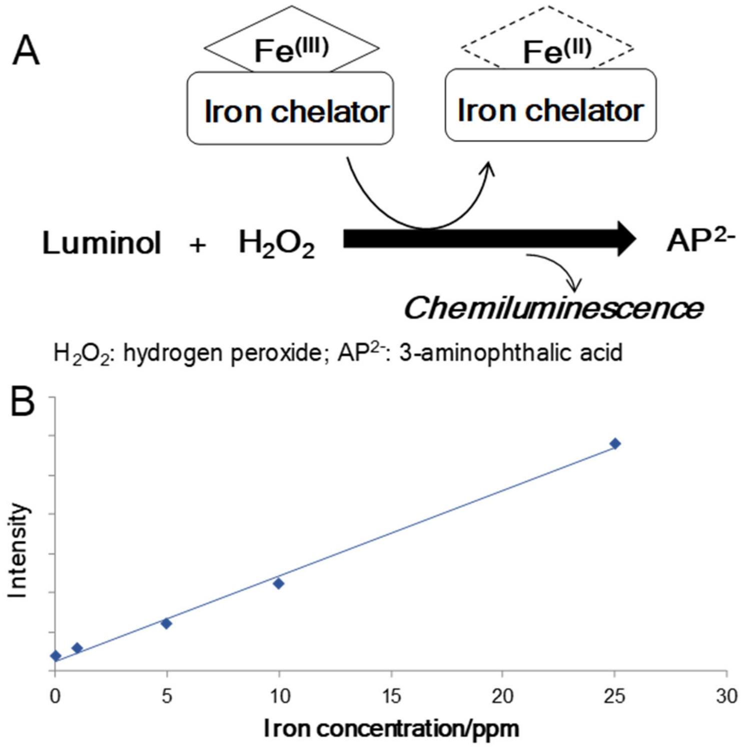

Figure S2. Mechanism of iron elution and its relationship with iron concentration (A) Due to the reaction of Fe(III) and luminol, chemiluminescence is released. (B) Iron concentration and the intensity of chemiluminescence are linearly related.

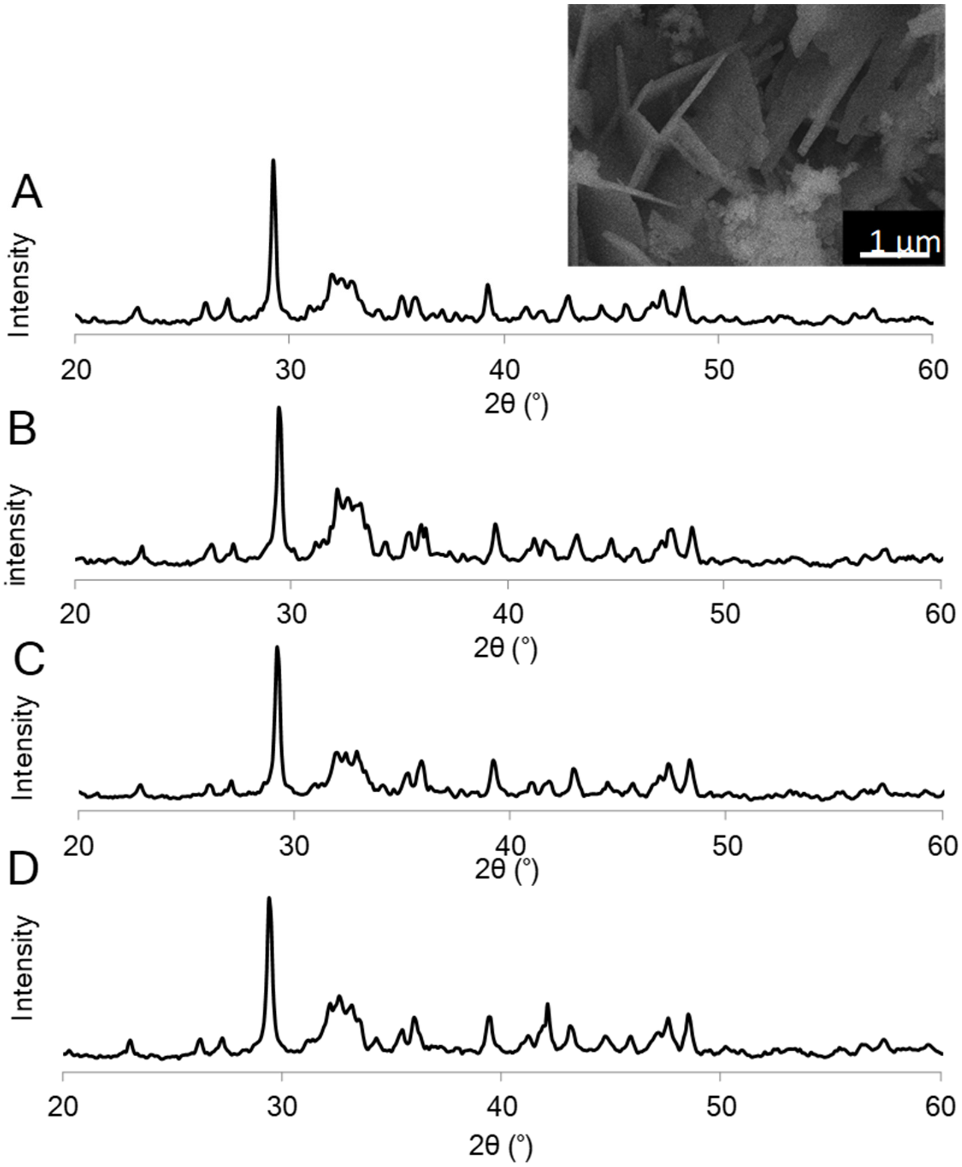

Figure S3. X-ray powder diffraction analysis of CSMS. (A) intact CSMS. The SEM image at upper right area showed the surface structure of intact CSMS. (B) CSMS incubated with sterilized medium (C) CSMS incubated with TO1A. (D) CSMS incubated with TO1B.

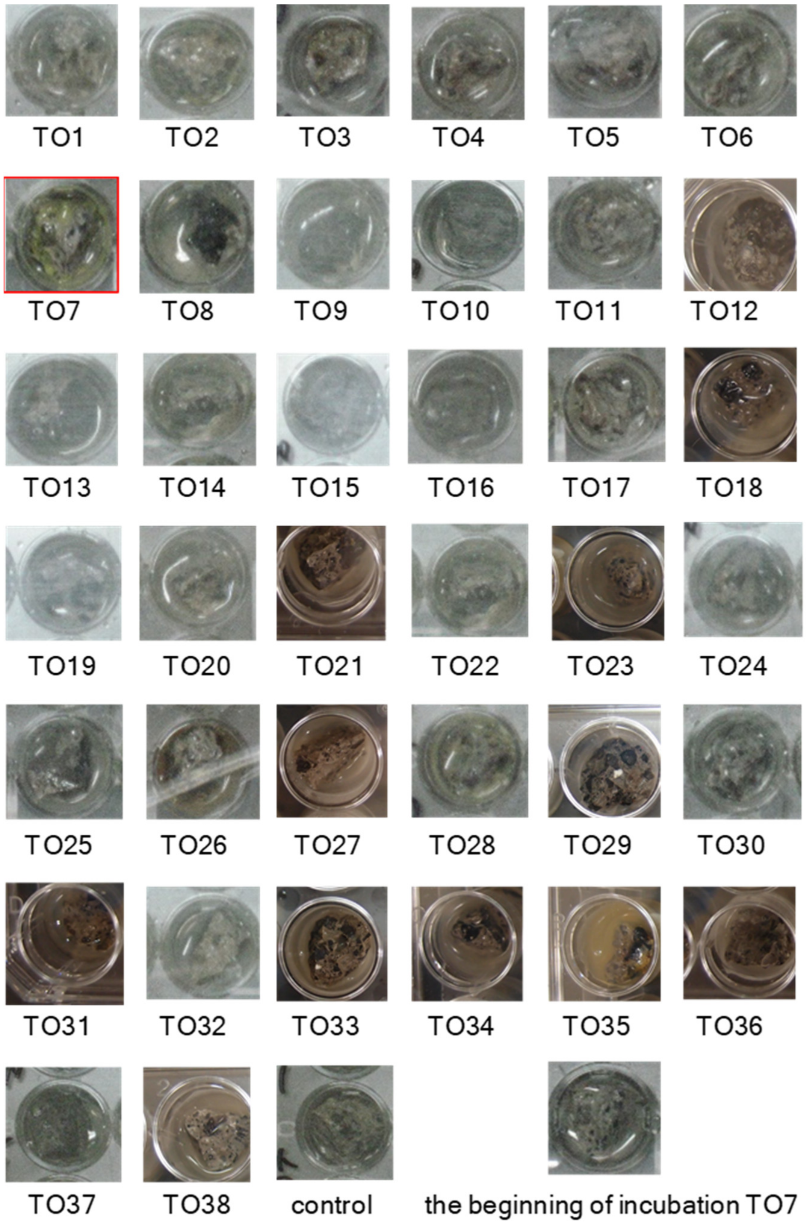

Figure S4. SSHM after incubation with TO strains. A large amount of yellow biofilm was formed on the surface of SSHMs incubated with TO7.

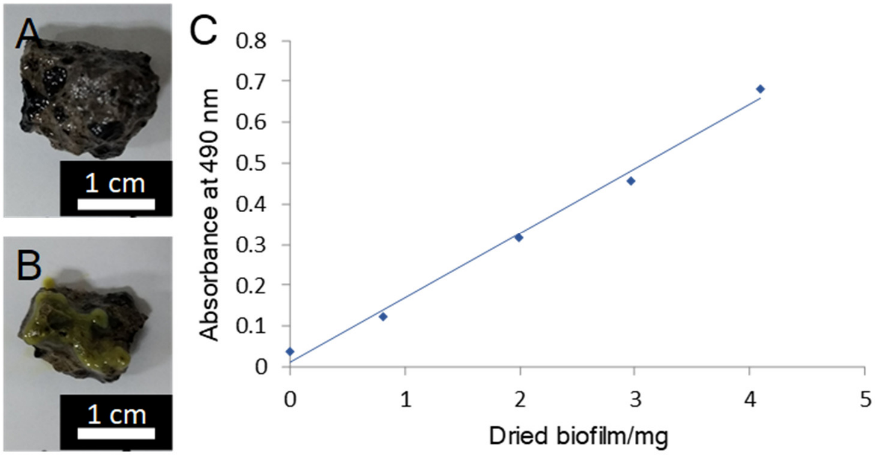

Figure S5. Biofilm and its absorbance at 490 nm. (A) SSHM without biofilm formation. (B) After incubation with TO7, yellow biofilm can be seen on the surface of the matrix. (C) The weight of dried biofilm and its absorbance at 490 nm were correlated.
